# Comparison of therapeutic effects of mesenchymal stem cells from umbilical cord and bone marrow in the treatment of type 1 diabetes

**DOI:** 10.1186/s13287-022-02974-1

**Published:** 2022-08-08

**Authors:** Wei Zhang, Qing Ling, Bin Wang, Kai Wang, Jianbo Pang, Jing Lu, Yan Bi, Dalong Zhu

**Affiliations:** 1 Department of Endocrinology, Affiliated Drum Tower Hospital, Medical School of Nanjing University, No. 321, Zhongshan Road, Nanjing, 210008 Jiangsu China; 2 Clinical Stem Cell Center, Affiliated Drum Tower Hospital, Medical School of Nanjing University, No. 321, Zhongshan Road, Nanjing, 210008 Jiangsu China

**Keywords:** Type 1 diabetes, Umbilical cord-derived mesenchymal stem cells, Bone marrow-derived mesenchymal stem cells, Cell transplantation

## Abstract

**Background:**

The therapeutic potential of mesenchymal stem cells (MSCs) in type 1 diabetes (T1D) has been demonstrated in both preclinical and clinical studies. MSCs that have been used in research on T1D are derived from various tissue sources, with bone marrow (BM) and umbilical cord (UC) tissues being the most commonly used. However, the influence of tissue origin on the functional properties and therapeutic effects of MSCs in T1D remains unclear. This study aimed to compare the therapeutic efficacy of UC-MSCs and BM-MSCs in a mouse model of T1D as well as in patients with T1D.

**Methods:**

In non-obese diabetic (NOD) mice, the development of diabetes was accelerated by streptozotocin injections. Thereafter, diabetic mice were randomized and treated intravenously with UC-MSCs, BM-MSCs or phosphate-buffered saline as a control. Blood glucose and serum insulin were measured longitudinally after transplantation. At 14 days post-transplantation, pancreatic tissues were collected to assess insulitis and the β-cell mass. Flow cytometry was performed to evaluate the composition of T lymphocytes in the spleen and pancreatic lymph nodes of the NOD mice. In our retrospective study of patients with T1D, 28 recipients who received insulin therapy alone or a single transplantation of UC-MSCs or BM-MSCs were enrolled. The glycaemic control and β-cell function of the patients during the first year of follow-up were compared.

**Results:**

In NOD mice, UC-MSC and BM-MSC transplantation showed similar effects on decreasing blood glucose levels and preserving β cells. The regulation of islet autoimmunity was examined, and no significant difference between UC-MSCs and BM-MSCs was observed in the attenuation of insulitis, the decrease in T helper 17 cells or the increase in regulatory T cells. In patients with T1D, MSC transplantation markedly lowered haemoglobin A1c (HbA1c) levels and reduced insulin doses compared to conventional insulin therapy. However, the therapeutic effects were comparable between UC-MSCs and BM-MSCs, and they also exerted similar effects on the endogenous β-cell function in the patients.

**Conclusion:**

In conclusion, both UC-MSCs and BM-MSCs exhibited comparable therapeutic effects on improving glycaemic control and preserving β-cell function in T1D. Considering their abundance and higher cell yields, UC-MSCs appear to be more promising than BM-MSCs in clinical applications.

*Trial registration* NCT02763423. Registered on May 5, 2016—Retrospectively registered, https://www.clinicaltrials.gov/.

## Introduction

Type 1 diabetes (T1D) is an autoimmune disease characterized by the selective destruction of insulin-producing β cells and the progressive loss of islet function. Despite the continued optimization of insulin therapy regimens, glycaemic control and overall outcomes are still far from satisfactory [1]. Blocking immune attack and preserving residual β-cell function remain challenging. Mesenchymal stem cells (MSCs) are multipotent cells derived from a variety of tissues, including bone marrow (BM), umbilical cord (UC), placenta, adipose tissue (AT) and pancreas [2]. Due to their multilineage differentiation potential and immunomodulatory capacities [3], MSCs have been widely studied in the treatment of degenerative and autoimmune diseases, including T1D [4, 5]. In preclinical studies of T1D, evidence has shown that MSCs can delay or even reverse the progression of diabetes in non-obese diabetic (NOD) mice [6–8]. The underlying mechanism is mainly associated with modulating autoimmune responses against islets. In addition to suppressing pathogenic CD8 + T cells, T helper 1 (Th1) cells and T helper 17 (Th17) cells [9, 10], MSCs can also promote regulatory T cells (Tregs) through paracrine effects, direct cell receptor interactions or mitochondrial transfer [11, 12]. Because islets are highly vascularized mini-organs, the promotion of angiogenesis by MSCs in islets is another potential mechanism in T1D [13, 14]. In addition, a novel effect of MSCs on reversing β-cell dedifferentiation may also contribute to alleviating T1D, but this phenomenon requires further investigation [15, 16].

Encouraging evidence from preclinical studies has promoted clinical translation. Over 15 trials are currently registered on *clinicaltrials.gov* to evaluate the safety and efficacy of MSCs in treating T1D. Although MSCs have shown tolerable safety, their ability to enhance islet function and reduce insulin dependence has not met expectations and has varied among published data [17–19]. In one randomized controlled trial (RCT), a single transplantation of autologous BM-MSCs showed obvious but limited improvement in β-cell function at the 1-year follow-up, with an approximately 5% increase in peak C-peptide levels in the MSC recipients compared to an approximately 15% decrease in those who received insulin alone. No improvement in glycaemic control or insulin dosage was observed [20]. In another RCT, patients who received allogenic UC-MSC transplantation showed significantly increased fasting C-peptide (FCP) levels (approximately 50% increase at 1-year follow-up), improved glycaemic control and reduced insulin doses even at 2 years after transplantation [21]. Although the treatment regimens of the two RCTs differed, one apparent difference is the tissue source of MSCs. The results of a meta-analysis suggested that UC-MSCs had a more beneficial effect than BM-MSCs on T1D [22]. However, because only one RCT was included for each type of MSC, the superiority of UC-MSCs requires further verification.

The heterogeneity of MSCs contributes to variations in therapeutic benefits and has thus led to increasing concerns in recent years [23]. The origin of tissue was the first determinant of the functional heterogeneity of MSCs to be recognized [2, 24, 25]. MSCs derived from different tissues vary in terms of their proliferative capacity, angiogenic potential and immunosuppressive ability [26–28]. However, few in vivo studies have systematically compared the therapeutic effects of MSCs on specific disorders. Currently, various sources of MSCs are used in clinical trials, with BM-MSCs and UC-MSCs being the most commonly used types [29]. Determining if differences exist between these two types of MSCs in treating T1D will provide important evidence for clinical translation.

In this study, we aimed to compare the therapeutic effects and mechanisms of UC-MSCs and BM-MSCs in a mouse model of T1D. In addition, a retrospective analysis was performed on patients with T1D who received UC-MSC or BM-MSC transplantation to further compare their safety and efficacy. For the first time, this study provided evidence at both the preclinical and clinical levels to support the application of UC-MSCs in the treatment of T1D.

## Materials and methods

### MSC isolation

MSCs were isolated, cultured and purified in Good Manufacturing Practice-certified facilities at the Clinical Stem Cell Center in the Affiliated Drum Tower Hospital, Medical School of Nanjing University, as described previously [9, 30]. Written informed consent was obtained from healthy human donors.

BM-MSCs were isolated from the BM aspirates of healthy relatives of the patients. Mononuclear cells were isolated from heparinized human BM samples (5 ml from each donor) by density-gradient centrifugation and cultured in Dulbecco’s Modified Eagle Medium (DMEM) GlutaMAX (Gibco, Grand Island, NY, USA) with 10% foetal bovine serum (FBS, Gibco, Grand Island, NY, USA) and 1% penicillin/streptomycin (Gibco, Grand Island, NY, USA) in a 5% CO_2_ incubator at 37 °C for 48 h. After non-adherent cells were removed, the remaining cells were exposed to fresh medium every 3–4 days until the cells were confluent. Subsequently, the cells were passaged.

UC-MSCs were isolated from the Wharton’s jelly of UC using the tissue explant method [30, 31]. Briefly, fresh UCs were obtained from informed healthy mothers in the maternity department after normal deliveries. A 10-cm piece of UC was cut into three equal pieces. Each piece was then cut into smaller pieces with a length of approximately 1 cm and washed thoroughly to remove blood and blood clots. The umbilical arteries, veins and adventitia were removed to obtain the Wharton’s jelly. Then, the Wharton’s jelly was dissected into small pieces of approximately 1 mm^3^. Properly cut tissue pieces were placed into a T75 cell culture flask at 0.5-cm intervals and incubated in a 5% CO_2_ incubator at 37 °C for 4 h [32]. Complete medium (DMEM GlutaMAX supplemented with 10% FBS and 1% penicillin/streptomycin) was slowly added for a prolonged period of culture, and the medium was changed every 3–4 days. When well-developed colonies of fibroblast-like cells appeared, the cells were trypsinized and transferred into a new flask for further expansion. Cells at passages 2–5 were harvested and cryopreserved for subsequent clinical and experimental use.

## MSC characterization

MSCs were identified based on three criteria according to the guidelines of the Mesenchymal and Tissue Stem Cell Committee of the International Society for Cellular Therapy [33]. For surface marker expression analysis, approximately 2 × 10^5^ cells at the fourth passage were harvested and resuspended in 100 μl of phosphate-buffered saline (PBS, HyClone, South Logan, UT, USA) and then stained with monoclonal antibodies (mAbs) labelled with FITC (CD34, CD14, CD45 and CD19) or PE (CD73, CD105, CD90 and HLA-DR) (BD Biosciences, San Jose, CA, USA). After being incubated in the dark for 15 min at room temperature, the cells were washed with 1 × PBS and resuspended in washing buffer for flow cytometry analysis. Cells were acquired on a BD Accuri C6 Plus (BD Biosciences, San Jose, CA, USA), and the data were analysed using FlowJo v10.4 (Treestar, Ashland, OR, USA). To examine multilineage differentiation, MSCs at the fourth passage were harvested and replated at a density of 1 × 10^5^ cells/well in a 6-well culture plate. When the cells reached 50–70% confluency, adipogenic differentiation medium (Gibco, Grand Island, NY, USA) or osteogenic differentiation medium (Gibco, Grand Island, NY, USA) was replaced to induce adipogenesis or osteogenesis, respectively. The differentiation medium was changed every 3 days. After 21 days, the cells were fixed in 4% formaldehyde (Servicebio, Wuhan, China) and stained with Oil Red O (Sigma-Aldrich, St. Louis, MO, USA) or Alizarin Red S (Sigma-Aldrich, St. Louis, MO, USA) to evaluate adipogenic or osteogenic differentiation, respectively.

### Mice and the diabetic model

Female NOD/Ltj mice at 10–12 weeks of age were purchased from the Animal Model Research Center of Nanjing University (Nanjing, China) and housed in a specific pathogen-free facility with a 12:12-h light/dark cycle and a constant temperature and humidity. All animal procedures were approved by the Animal Ethics Committee of the Affiliated Drum Tower Hospital, Medical School of Nanjing University (Approval Number: 20191205).

NOD/Ltj mice were injected intraperitoneally with 40 mg/kg streptozotocin (STZ, Sigma-Aldrich, St. Louis, MO, USA) for five consecutive days to accelerate the development and progression of diabetes. Individual mice with blood glucose levels ≥ 13.9 mmol/L for two consecutive days were diagnosed with diabetes [9, 34, 35]. Mice that failed to develop diabetes and those whose blood glucose levels exceeded the glucometer range were excluded to reduce bias.

### Treatment of NOD mice

Diabetic mice were randomized and treated intravenously with 300 μl of PBS alone as the control group, 1 × 10^6^ BM-MSCs resuspended in 300 μl of PBS as the BM-MSC group and 1 × 10^6^ UC-MSCs in 300 μl of PBS as the UC-MSC group. After treatment, body weights and random blood glucose levels were measured twice a week, and the serum insulin levels were examined weekly by ELISA using an Ultrasensitive Mouse Insulin ELISA kit (Mercodia AB, Uppsala, Sweden). At two weeks post-treatment, mice in each group were killed, and their tissues were dissected for subsequent analysis.

### Histology and immunohistochemistry

Pancreatic tissues from the mice were fixed in 10% neutral buffered formalin (Servicebio, Wuhan, China) and embedded in paraffin. Then, 3-μm sections were cut using a microtome (Leica Microsystems, Wetzlar, Germany), and four sections with a 150-μm gap for each pancreas were chosen and measured to prevent double counting of the same islet [34]. The sections were deparaffinized, rehydrated and stained with haematoxylin and eosin (H&E, Servicebio, Wuhan, China). Four mice in each group were used to determine insulitis, and four tissue slices were prepared from each mouse. Insulitis was evaluated on a 0–4 scale (0, no insulitis; 1, leukocyte infiltration ≤ 25%; 2, leukocyte infiltration > 25%, but ≤ 50%; 3, leukocyte infiltration > 50%; and 4, leukocyte infiltration 100% and β-cell destruction) [36, 37]. At least 20 islets from the pancreatic tissue of each mouse were evaluated. Insulitis scoring was performed by two researchers who were blinded to the experimental conditions and who independently reviewed the slide scans.

The insulin content in the islet β cells was determined by immunohistochemistry. Briefly, pancreatic tissue sections were deparaffinized, rehydrated and treated with 3% H_2_O_2_ (Servicebio, Wuhan, China) in methanol (Sinopharm Chemical Reagent Co., Ltd, Wuhan, China) for 25 min to inactivate endogenous peroxidase activity. The sections were subjected to antigen retrieval and treated with 3% bovine serum albumin (Servicebio, Wuhan, China) for 30 min. Subsequently, the sections were incubated with mouse anti-insulin antibodies (1:1000; Servicebio, Wuhan, China), and after being washed, the bound antibodies were detected with horseradish peroxidase-conjugated goat anti-mouse IgG (1:200; Servicebio, Wuhan, China) and visualized with diaminobenzidine (Servicebio, Wuhan, China). The level of insulin staining in individual images was measured and presented as the ratio of the positively stained areas in the islets to the total islet areas using Image-Pro Plus 6.0 (Planetron, Tokyo, Japan). At least 20 islets from the pancreatic tissue of each mouse were analysed.

### Flow cytometry

Single-cell suspensions were isolated from mouse spleens and pancreatic lymph nodes (PLNs) and stimulated with Leukocyte Activation Cocktail (BD Biosciences, San Jose, CA, USA) for 4 h for subsequent intracellular cytokine analysis. Fc receptors were blocked with mouse FcR Blocking Reagent (Miltenyi Biotec, Bergisch Gladbach, Germany) for 10 min at 4 °C, followed by incubation with fluorochrome-conjugated antibodies against cell surface markers for 15 min at room temperature. Cells were also stained with Fixable Viability Stain 780 (BD Biosciences, San Jose, CA, USA). After extracellular staining, the cells were fixed by a fixation/permeabilization kit (BD Biosciences, San Jose, CA, USA) according to the manufacturer’s instructions and subsequently stained with specific antibodies for intracellular cytokines or intranuclear transcription factors for 45 min at 4 °C. Multiparameter flow cytometry was performed with the following mAbs: FITC anti-CD3e (clone 145-2C11, BD Biosciences, San Jose, CA, USA), AF700 anti-CD4 (clone RM4-5, BD Biosciences, San Jose, CA, USA), V450 anti-CD8 (clone 53–6.7, BD Biosciences, San Jose, CA, USA), BV786 anti-IL-17 (clone TC11-18H10, BD Biosciences, San Jose, CA, USA) and PE-Cy7 anti-FOXP3 (clone 145-2C11, eBioscience, San Diego, CA, USA). Cells were acquired on a BD FACSAriaIII (BD Biosciences, San Jose, CA, USA). The data were analysed by FlowJo v10.4 (Treestar, Ashland, OR, USA).

### Patients

In total, 28 patients with T1D were recruited from the Department of Endocrinology of the Affiliated Drum Tower Hospital, Medical School of Nanjing University (Nanjing, China), from March 2009 to July 2012. T1D was diagnosed based on the following clinical features: positive for at least one of the following—glutamic acid decarboxylase antibody (GADA), protein tyrosine phosphatase antibody (IA-2A), islet cell antibody (ICA) and insulin autoantibody (IAA), and/or an FCP ≤ 200 pmol/L. Written informed consent was obtained from all participants and/or guardians and the MSC donors. This study was approved by the Ethics Committee of our hospital.

Eligible participants were aged 8–60 years with insulin requirements since the diagnosis of T1D. Exclusions included cardiorespiratory insufficiency, failure of one or more organs, infection with human immunodeficiency virus or hepatitis virus, pregnancy, underlying haematologic, rheumatic, psychiatric or malignant disease or previous treatment with immunosuppressants. Participants who consented to receive MSC therapy were assigned to the intervention group, while the others were assigned to the control group.

### Treatment and follow-up

All participants received routine T1D treatment, including lifestyle management and intensive insulin therapy. T1D education was offered to all subjects, who were instructed to self-monitor their blood glucose and adjust their insulin dosage accordingly. Participants in the MSC-treated group were intravenously infused with BM-MSCs or UC-MSCs at a dose of 1 × 10^6^ cells/kg body weight in addition to regular insulin treatments. All participants were followed up for one year post-treatment. Blood samples were obtained before and one year after treatment for standard measurements of plasma haemoglobin A1c (HbA1c) and C-peptide during a standardized meal. Individual patients who achieved a 10% increase from baseline in the FCP and/or postprandial C-peptide (PCP) levels were considered to be in clinical remission. The incidence and severity of adverse events were assessed and recorded throughout the study.

### Statistical analysis

All statistical analyses were performed using SPSS version 23.0 (IBM, Armonk, NY, USA). The data are presented as the mean ± standard error of the mean (SEM) unless otherwise stated. Differences between two unpaired groups were compared by Student’s t test or the Mann–Whitney U test. Statistical variations between three groups within an experiment were analysed by one-way ANOVA, followed by the post hoc Fisher’s least significant difference test. Categorical variables were compared by Chi-square analysis or Fisher’s exact test. A two-tailed *P* value of < 0.05 was considered statistically significant.

## Results

### Characterization of BM-MSCs and UC-MSCs

**Fig. 1:**
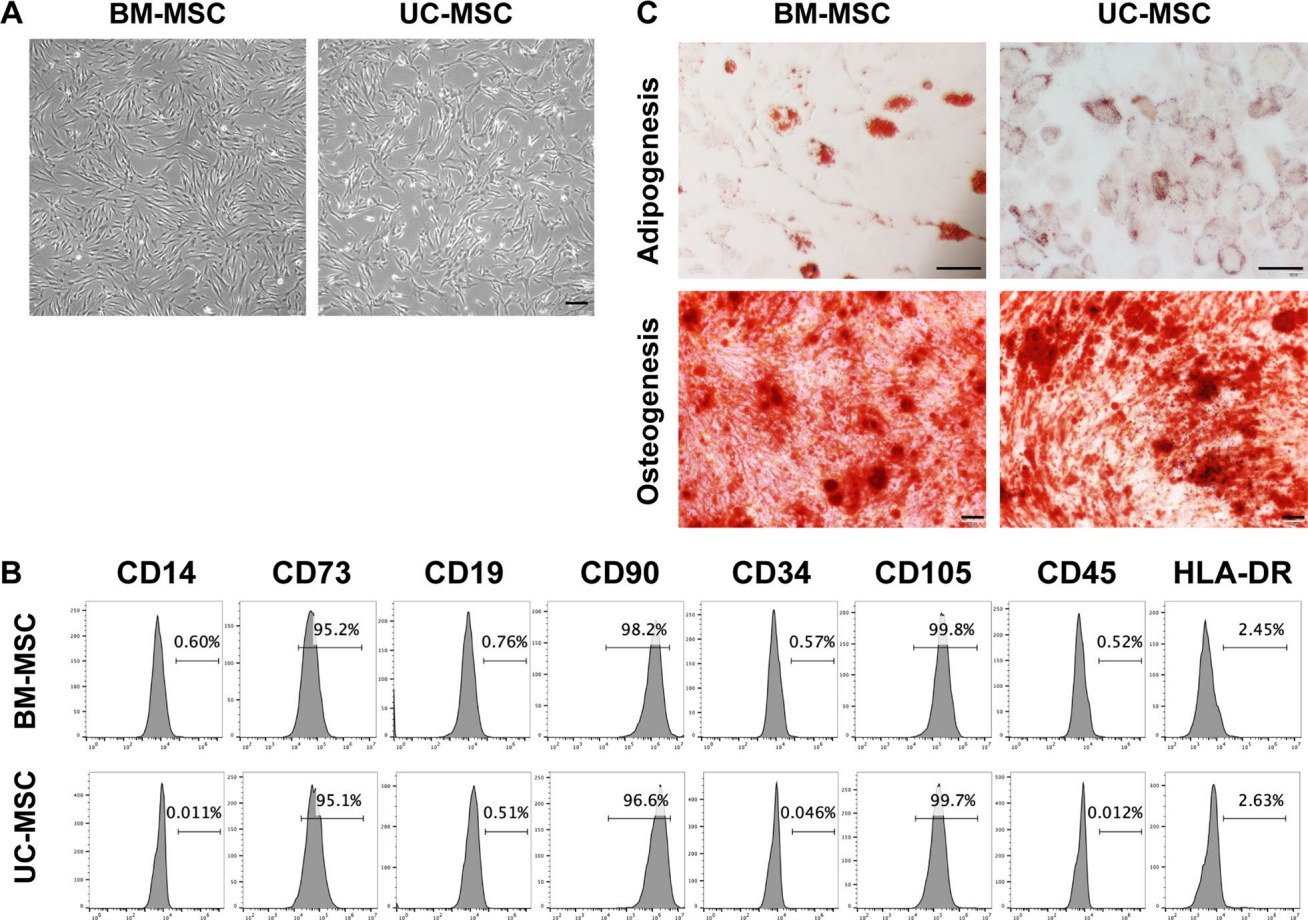
Characterization of BM-MSCs and UC-MSCs. **A** Representative micrographs of BM-MSCs and UC-MSCs observed under light microscopy. Scale bar, 200 μm. **B** Expression of CD14, CD73, CD19, CD90, CD34, CD105, CD45 and HLA-DR in BM-MSCs and UC-MSCs analysed by flow cytometry. **C** Representative micrographs of adipogenesis identified by Oil Red O staining, and osteogenesis identified by Alizarin Red staining of BM-MSCs and UC-MSCs. Scale bar, 100 μm

We first characterized the phenotypes of BM-MSCs and UC-MSCs by measuring their morphology, specific surface marker expression and multilineage differentiation abilities. Both BM-MSCs and UC-MSCs exhibited the typical morphology of fibroblasts and displayed a high capacity to adhere to the plastic disc (Fig. 1A). Both types of cells were strongly positive (> 95%) for the MSC-specific surface markers CD73, CD90 and CD105 but were negative (< 3%) for CD14, CD19, CD34, CD45 and HLA-DR (Fig. 1B). Moreover, these cells could successfully differentiate into adipocytes and osteocytes under standard cell induction conditions (Fig. 1C).

### MSCs decrease blood glucose levels in NOD mice

**Fig. 2:**
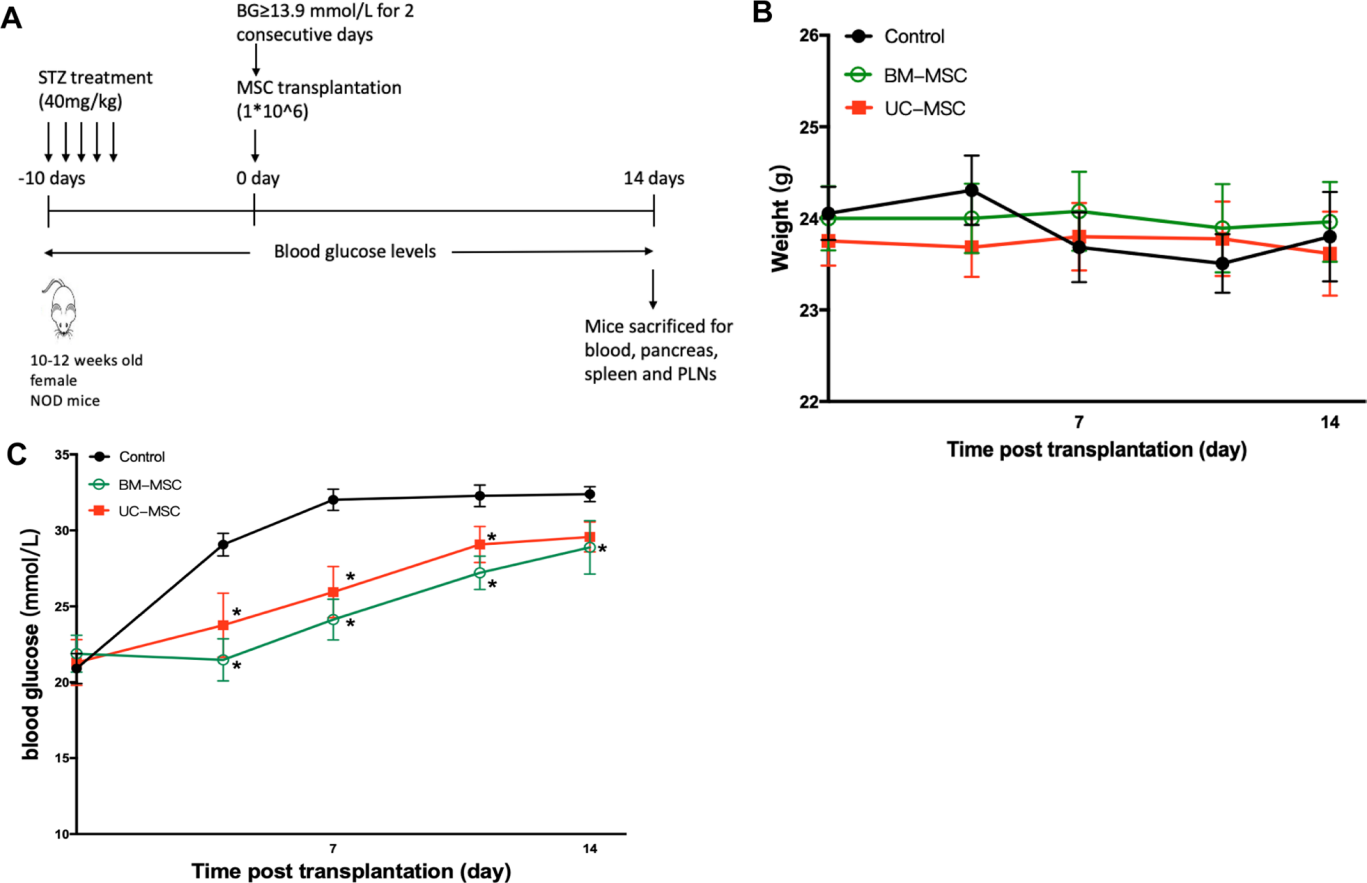
Effects of BM-MSCs or UC-MSCs infusion on body weights and blood glucose levels in NOD mice. **A** The treatment schedule for STZ and MSC transplantation. Body weights (**B**) and blood glucose levels (**C**) were measured twice a week after MSC transplantation for 14 days (*N* = 13 in each group). Data are presented as mean ± SEM. *P < 0.05 versus the control group

Prediabetic female NOD mice were injected multiple times with low doses of STZ to accelerate the development of T1D. When the mice developed diabetes, they were randomized to groups and administered PBS, BM-MSCs or UC-MSCs. Body weights and blood glucose levels were monitored longitudinally for two weeks (Fig. 2A). No significant differences were observed between the body weights of the control and MSC-treated mice (Fig. 2B). The blood glucose levels in the MSC-treated mice were relatively stable during the 14 days after transplantation compared to those of the mice in the control group, which increased rapidly during the first week and remained high. The blood glucose levels in the BM-MSC group and the UC-MSC group were significantly lower than those in the control group at 4, 7, 11 and 14 days post-transfusion. However, no significant difference in blood glucose between the two types of MSCs was observed (Fig. 2C).

### MSCs preserve β-cell function in NOD mice

**Fig. 3:**
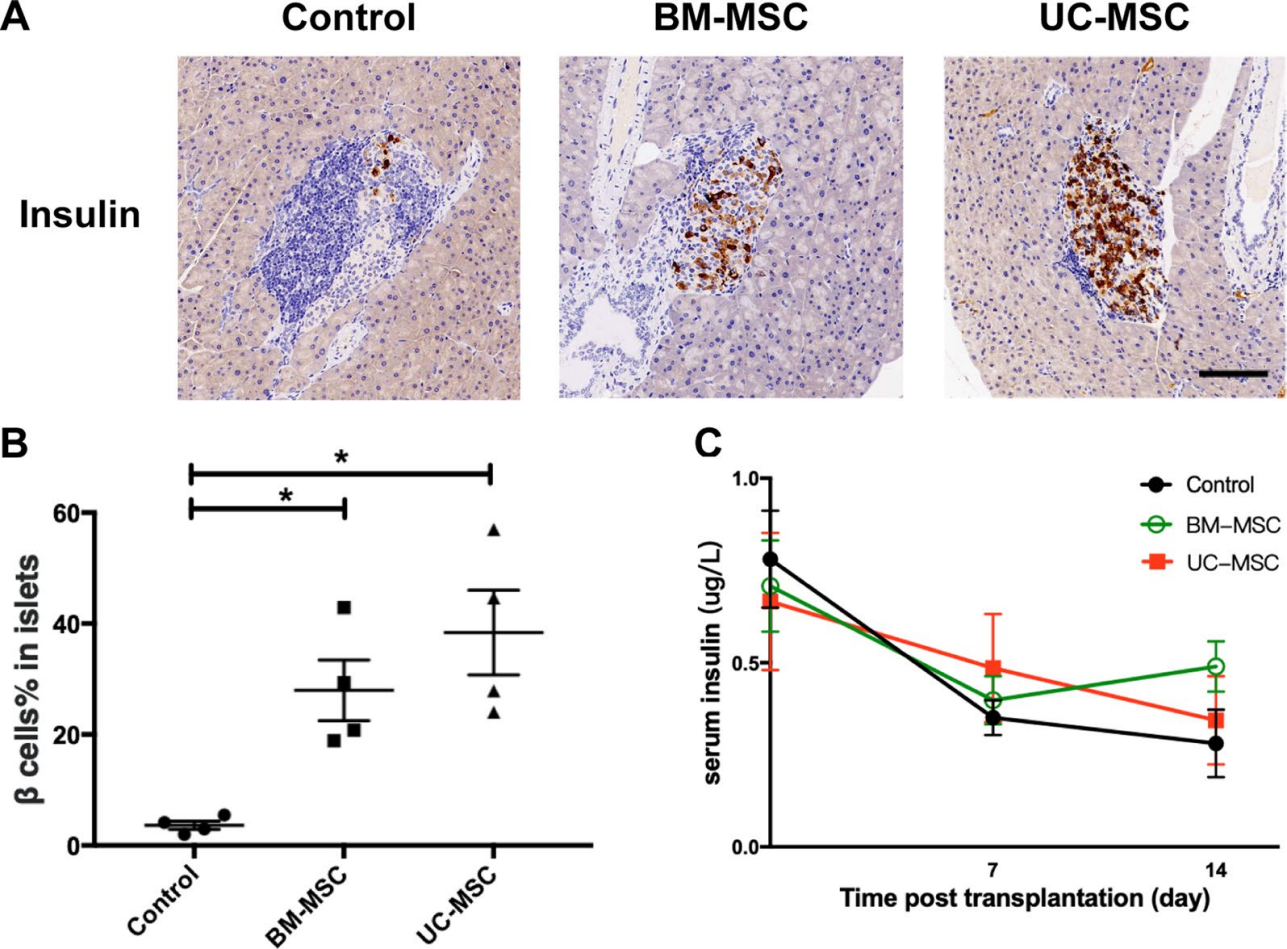
Effects of BM-MSCs or UC-MSCs infusion on β-cell mass and insulin production. **A** Representative micrographs of insulin immunohistochemical staining showing β cells preserved in islets at 14 days post-transplantation. Scale bar, 100 μm. **B** The insulin-positive staining areas (%) in pancreatic islets were quantified using the Image-Pro Plus 6.0. Four slides per mouse (four mice per group) were analysed, and at least 20 islets from the pancreatic tissue of each mouse were evaluated. **C** Random serum insulin concentration (*N* = 4–5). Data are presented as mean ± SEM. *P < 0.05 versus the control group

To investigate the effects of BM-MSC and UC-MSC infusion on β-cell function, an immunohistochemical analysis of the islets was performed 14 days post-MSC transplantation, and serum insulin levels were measured. The percentage of insulin-positive areas in the BM-MSC group and the UC-MSC group was significantly higher than that in the control group (Fig. 3A, B). All three groups showed a decreasing tendency in the levels of serum insulin after the time of treatment. The absolute level of serum insulin was slightly higher in the BM-MSC group and the UC-MSC group than in the control group at 7 days and 14 days post-transfusion, but the difference was not significant (Fig. 3C).

In a further comparison between the two types of MSCs, the β-cell mass in the UC-MSC group was slightly higher than that in the BM-MSC group at 14 days post-transplantation (Fig. 3A, B). The UC-MSC-treated mice showed slightly higher levels of serum insulin on day 7, while the BM-MSC-treated mice showed higher levels of serum insulin on day 14, although the differences were not significant, suggesting that BM-MSCs and UC-MSCs had a similar ability to preserve β-cell function in islets.

### Immunomodulatory effects of MSCs on NOD mice

**Fig. 4:**
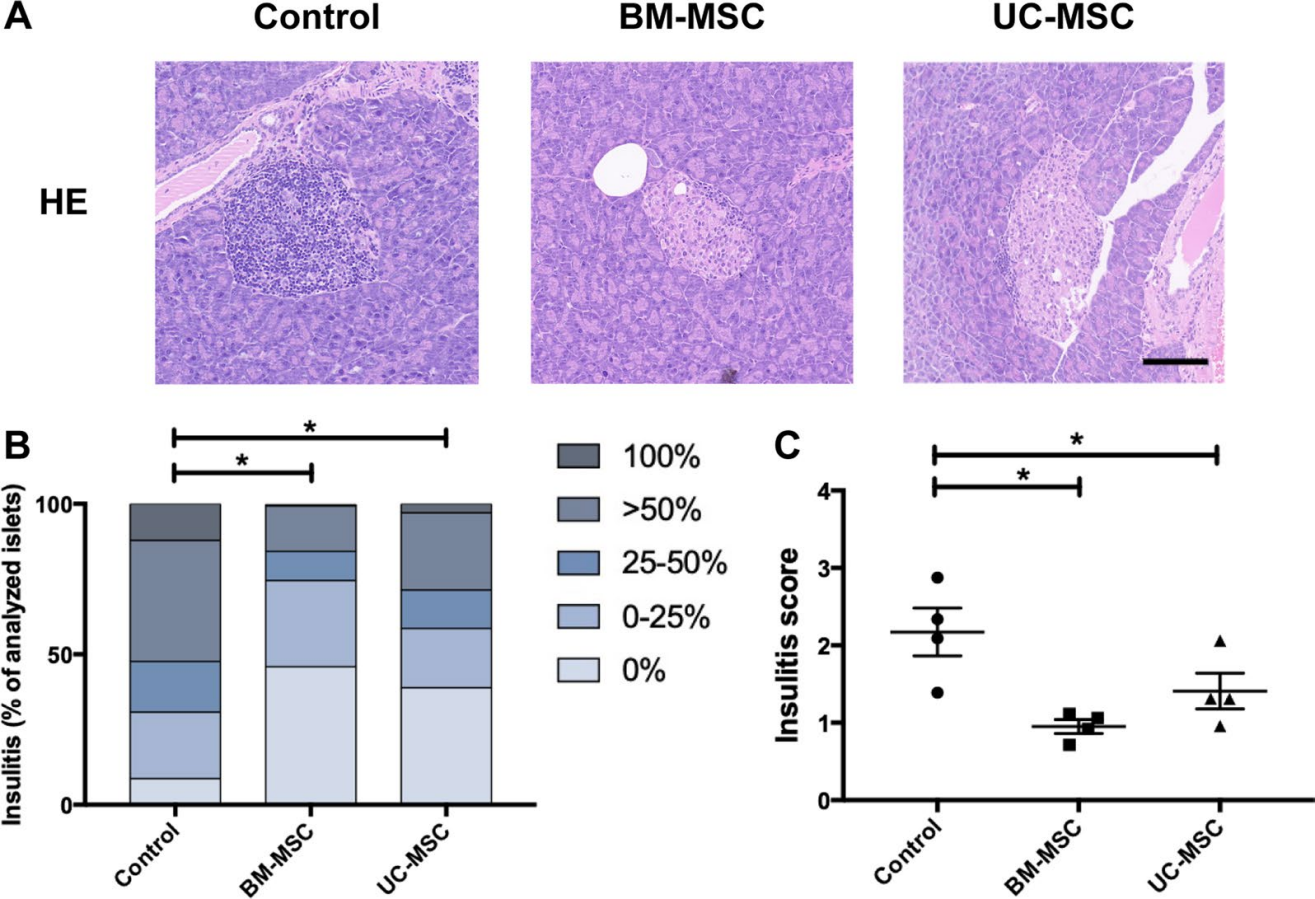
Effects of BM-MSCs or UC-MSCs infusion on insulitis in NOD mice. **A** Representative micrographs of H&E staining of pancreas at 14 days post-transplantation. Scale bar, 100 μm. **B** The percentage of islets in each of the infiltration categories (score 0, no insulitis; score 1, leukocyte infiltration ≤ 25%; score 2, leukocyte infiltration > 25%, but ≤ 50%; score 3, leukocyte infiltration > 50%; score 4, leukocyte infiltration 100% and β-cell destruction). **C** Insulitis score in pancreatic islets. Four slides per mouse (four mice per group) were analysed, and at least 20 islets from the pancreatic tissue of each mouse were evaluated. Data are presented as mean ± SEM. *P < 0.05 versus the control group

T1D is caused by the infiltration of immune cells (mainly autoreactive T cells) into pancreatic islets and autoimmune-mediated β-cell destruction. Therefore, we further evaluated the immunomodulatory effects of the two kinds of MSCs. In the control mice, only 8.7% of islets were free from insulitis, and over 50% of islets exhibited severe insulitis (> 50% infiltration) or were completely infiltrated with leukocytes. In contrast, more than 40% of islets in the BM-MSC-treated or UC-MSC-treated mice were free from infiltration, and only approximately 20% of islets exhibited severe insulitis (Fig. 4A, B). Consistently, the insulitis scores of the mice treated with BM-MSCs or UC-MSCs were significantly lower than that of the control mice (1.0 ± 0.1 or 1.4 ± 0.2 vs. 2.2 ± 0.3, *P* < 0.05, Fig. 4C). In a further comparison between the two types of MSCs, the proportion of islets that were free of infiltration in the BM-MSC group was slightly higher than that in the UC-MSC group (45.9% vs. 38.9%, *P* > 0.05), and the proportion of islets with over 50% infiltration was relatively lower in the BM-MSC group than in the UC-MSC group (15.6% vs. 28.6%, *P* > 0.05, Fig. 4B). Overall, the insulitis score of the BM-MSC group was slightly lower than that of the UC-MSC group (1.0 ± 0.1 vs. 1.4 ± 0.2, *P* > 0.05, Fig. 4C). However, no significant differences were observed in the above comparisons between the two groups, indicating that BM-MSCs and UC-MSCs possessed a comparable ability to reduce insulitis in the NOD mice.

**Fig. 5:**
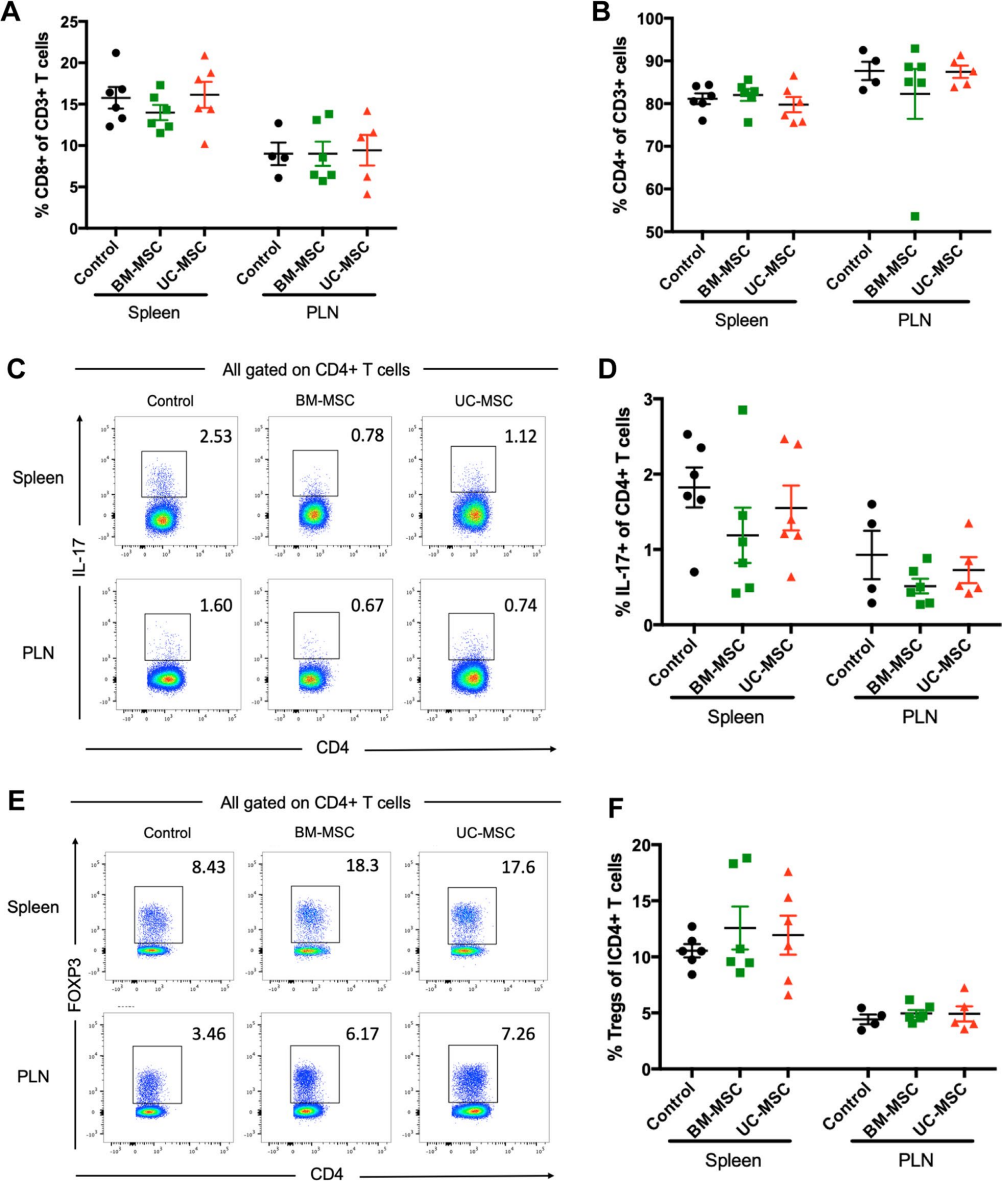
Effects of BM-MSCs or UC-MSCs infusion on T cell proportion in NOD mice. Percentage of CD8 + T cells (**A**) and CD4 + T cells (**B**) in gated CD3 + T cell population in the spleen and PLNs from NOD mice killed at 14 days post-treatment. Representative cytofluorometric dot plots (**C**) and summary data (**D**) of Th17 in gated CD4 + T cell population in the spleen and PLNs from NOD mice killed at 14 days post-treatment. Representative cytofluorometric dot plots (**E**) and summary data (**F**) of Tregs in gated CD4 + T cell population in the spleen and PLNs from NOD mice killed at 14 days post-treatment (*N* = 4–6). PLN, pancreatic lymph node

T cells play an important role in the development of T1D. An imbalance in pathogenic T cells and Tregs contributes to the pathogenesis of T1D. Importantly, Th17 cells are involved in the pathogenesis of autoimmune diabetes both in patients with T1D [38] and in NOD mice [39]. Therefore, we examined the frequencies of T cells in the spleen and local PLNs by flow cytometry. The frequencies of CD4 + T cells or CD8 + T cells in the spleen and PLNs showed no difference between the control mice and the MSC-treated mice (Fig. 5A, B). However, in both MSC-treated groups, the frequencies of Th17 cells (IL-17 + CD4+) in the spleen and PLNs appeared to be lower, while splenic Tregs (FOXP3 + CD4+) were relatively higher compared to those in the control group, although the differences were not significant (Fig. 5C–F). There was no significant difference in the immunomodulatory effects of BM-MSCs and UC-MSCs on the NOD mice.

### MSC transplantation improves glycaemic control and reduces insulin dosage in patients with T1D

A total of 14 patients with T1D received MSC transplantation in our department from March 2009 to July 2012; 4 patients received BM-MSC transfusion, while 10 patients received UC-MSC transfusion. Moreover, 14 patients who received regular insulin treatment alone were enrolled as the control group. The mean age of all the participants was 18.3 ± 9.3 years, and the duration of time from diagnosis of T1D to enrolment in this study ranged from 0 to 12 months. All the participants presented obvious symptoms of hyperglycaemia at the onset of T1D, and 92.9% of them were complicated with diabetic ketosis or diabetic ketoacidosis. The mean HbA1c level was 9.9 ± 3.0%, and the mean body mass index was 17.7 ± 2.7 kg/m^2^. The demographic and baseline characteristics were similar between the MSC-treated and control groups (Table 1). No adverse events were observed in any of the participants during the 1-year follow-up.

**Table 1:** Baseline characteristics of the participants

	Control group (*n* = 14)	MSC-treated group (*n* = 14)	*P* value
Gender (M/F)	3/11	3/11	1.000
Age (year)	17 (15, 24)	15 (10, 24)	0.299
Duration (month)	3.8 (0.6, 8.0)	1.0 (0, 3.3)	0.171
DKA/DK history (case)	12	14	0.481
BMI (kg/m^2^)	18.0 ± 3.3	17.3 ± 2.0	0.497
HbA1c (%)	9.1 ± 2.4	10.6 ± 3.4	0.197
Islet autoantibody (case)			1.000
Negative	2	1	
1 positive	4	6	
≥ 2 positive	8	7	
FCP (pmol/L)	247.9 ± 129.2	220.9 ± 138.5	0.599
PCP (pmol/L)	564.3 ± 334.0	677.3 ± 438.1	0.450
Insulin doses (U/kg/d)	0.47 ± 0.15	0.62 ± 0.32	0.123

Data are shown as number (*n*), mean ± standard deviation or median (interquartile range)

*MSC* mesenchymal stem cell, *DK* diabetic ketosis, *DKA* diabetic ketosis acidosis, *BMI* body mass index, *HbA1c* haemoglobin A1c, *FCP* fasting C-peptide, *PCP* postprandial C-peptide

**Fig. 6:**
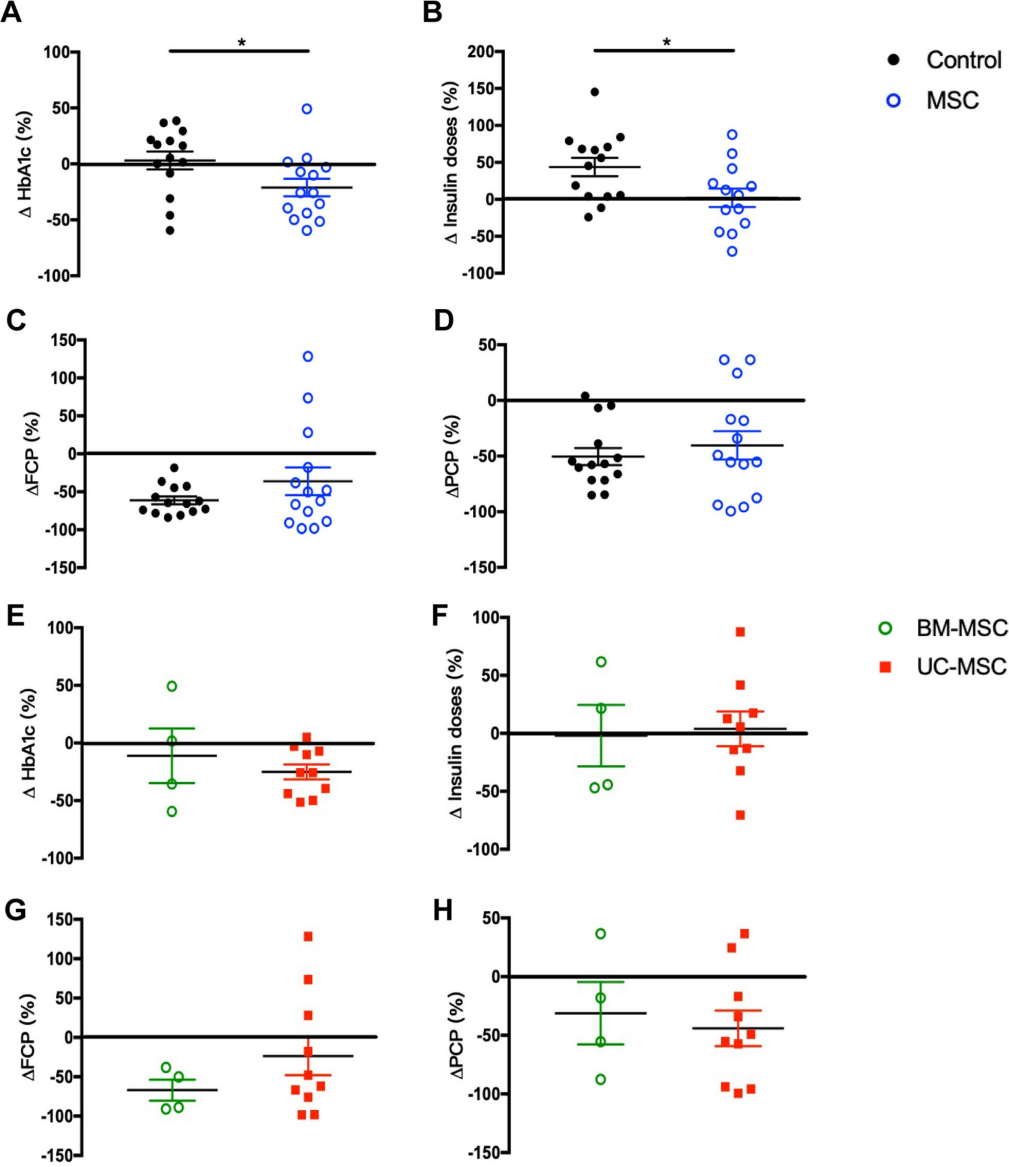
Change in HbA1c, exogenous insulin dosage and C-peptide in patients with T1D at 1-year follow-up. Rate of change in HbA1c (**A**), doses of daily insulin (**B**), FCP (**C**) and PCP (**D**) of the control group and MSC-treated group between baseline and 1-year follow-up. Rate of change in HbA1c (**E**), doses of daily insulin (**F**), FCP (**G**) and PCP (**H**) of the BM-MSC subgroup and UC-MSC subgroup between baseline and 1-year follow-up. Data are presented as mean ± SEM. *P < 0.05 versus the control group. FCP, fasting C-peptide; PCP, postprandial C-peptide

At the end of the 1-year follow-up, 4 recipients in the MSC-treated group maintained clinical remission (4/14, 28.6%), while no one in the control group achieved clinical remission (*P* = 0.098). The HbA1c levels in the MSC-treated group significantly decreased, while a slight increase was observed in the control group (*P* = 0.040, Fig. 6A). In addition, the daily insulin doses in the MSC-treated group were significantly lower than those in the control group (*P* = 0.027, Fig. 6B). The C-peptide levels decreased in all patients in the control group during the 1-year follow-up, while 4 patients in the MSC-treated group showed increased FCP or PCP levels. The average level of FCP at 1 year post-transplantation decreased by 61.2% of the baseline level in the control group, whereas patients who were treated with MSCs only showed a 36.1% decrease in the baseline level. Furthermore, the average level of PCP decreased by 50.4% in the control group and 40.4% in the MSC-treated group. Although the MSC-treated group showed attenuated reductions in FCP and PCP levels compared with the control group, no significant differences were observed (Fig. 6C, D).

To determine if there was a difference in the therapeutic efficacy of BM-MSCs and UC-MSCs, we performed subgroup analysis based on the type of infused MSCs. The demographic characteristics and baseline levels of HbA1c, FCP and PCP were comparable between the BM-MSC and UC-MSC groups. At the end of the 1-year follow-up, 1 recipient in the BM-MSC group (1/4, 25.0%) and 3 recipients in the UC-MSC group (3/10, 33.3%) achieved clinical remission (*P* = 1.000). The level of HbA1c at 1 year post-transplantation decreased by 11.1% of the baseline level in patients who were treated with BM-MSCs, whereas patients who were treated with UC-MSCs showed a 25.1% decrease in the baseline level. However, no significant difference was observed between the two groups in terms of the decline of the HbA1c levels (Fig. 6E). Although the patients treated with UC-MSCs showed a smaller decrease in the FCP levels (Fig. 6G) and a larger decrease in the PCP levels (Fig. 6H) than those treated with BM-MSCs, no significant differences were observed. There was no difference in the change in the daily insulin dose between the two groups (Fig. 6F).

## Discussion

This study evaluated and directly compared the therapeutic potential of UC-MSCs and BM-MSCs in NOD mice and in patients with T1D. MSCs derived from both tissue sources significantly improved glycaemic control and may have helped to preserve β-cell function. Moreover, the efficacy of UC-MSCs was non-inferior compared to that of BM-MSCs in the treatment of T1D.

MSCs were first isolated and characterized from BM [40], and thus, BM-MSCs are the most extensively studied type of MSC. Previous evidence showing that MSCs can delay or reverse T1D in animal models was largely based on studies using BM-MSCs [6, 41]. Since the 2000s, MSCs have been isolated from various other sources, and some of these cells have also been applied in studies of T1D. For instance, MSCs from the gingiva, menstrual blood or AT were reported to be effective in decreasing blood glucose levels, reducing insulitis and improving insulin levels in mouse models of T1D [14, 34, 42]. AT-MSCs along with vitamin D were demonstrated to decrease HbA1c levels and insulin requirement in patients with recent-onset T1D [43]. Furthermore, Lv et al. reported that depletion of diabetic gut microbiota resistance enhanced the effect of AT-MSCs in the treatment of T1D [34]. Nevertheless, the origin of tissues determines the intrinsic biological heterogeneity of MSCs. The extent to which the origin of MSC tissues influences their therapeutic efficacy in T1D remains unknown.

Recent studies of other diseases support the rationale of comparing the therapeutic effects of MSCs from different sources. For example, MSCs derived from dental pulp were shown to be more effective than BM-MSCs in cerebral ischaemic injury due to their better angiogenic effects [44]. BM-MSCs are more promising than UC-MSCs for the treatment of osteoarthritis because of their superior chondrogenic potential [45]. However, although MSCs derived from periodontal ligament (PDL-MSCs) showed a higher osteogenic potential and UC-MSCs secreted higher levels of extracellular matrix and had superior anti-inflammatory abilities, both types of MSCs generated similar effects in terms of the promotion of periodontal regeneration in periodontitis [46]. Specific MSC types may be suitable for the treatment of different diseases. Therefore, to transfer MSC therapy from bench to bedside, comparative studies of therapeutic competency are required to determine the appropriate tissue origin of MSCs for the treatment of T1D.

BM-MSCs and UC-MSCs are the most commonly used MSCs among registered clinical trials of T1D. Although more preclinical evidence is based on BM-MSCs, UC-MSCs exhibit several advantages and are considered to be promising candidates [47]. UC-MSCs, which are harvested from UCs that are normally discarded as medical waste, rarely raise ethical issues. The collection of UCs is an ex vivo and non-invasive process without risks of discomfort or infection; thus, it is a more acceptable procedure for donors. Moreover, UC-MSCs exhibit stronger proliferative capacities than BM-MSCs in vitro [48, 49], which leads to higher cell yields and a sufficient supply for clinical demands. Moreover, in vitro expanded UC-MSCs are a well-organized population with limited heterogeneity, which may be helpful for the standardization of MSC products [50]. It is worth noting that the UC-MSCs used in the current study were derived from the Wharton’s jelly rather than the blood of UCs. UC blood comprises numerous types of stem cells, but they were demonstrated to be less effective in treating T1D than UC-MSCs [51].

Consistent with previous reports [9, 21], this study showed that both UC-MSCs and BM-MSCs were effective in the treatment of T1D. Infusing the NOD mice with MSCs from either source significantly decreased blood glucose levels and preserved β-cell function. While evaluating the residual β-cell function of the NOD mice, the levels of serum insulin decreased in all groups, which was inconsistent with the corresponding histological results. This inconsistency is probably due to the measurement of random serum insulin, and an intraperitoneal glucose tolerance test should be used as a more objective potency assay in the future. In patients with T1D, transfusion with either type of MSC significantly decreased HbA1c levels and reduced the insulin dose. Although patients in the MSC-treated group presented an attenuation of the decrease in the C-peptide levels compared with those in the control group, we did not observe a significant improvement in the β-cell function as reported by Hu J et al. [21]. This outcome is probably due to disparities in the study design, the recruited participants, the preparation of MSCs and, most obviously, the transfusion regimen. Hu et al. repeatedly transplanted allogenic UC-MSCs, while we performed a single transplantation. According to our previous study on NOD mice and a recently published clinical report, increased courses of MSC transfusion may enhance the therapeutic efficacy in the treatment of T1D [9, 52], which is another point that is worthy of optimization.

Although UC-MSCs and BM-MSCs are both effective in the treatment of T1D, no evidence is available to determine which type of MSC has a superior therapeutic effect. Previous studies have demonstrated that UC-MSCs were more potent in inhibiting lymphocyte proliferation than BM-MSCs in vitro [27, 53], indicating that UC-MSCs have a stronger immunosuppressive ability. However, Amable et al. reported that UC-MSCs secreted higher concentrations of chemokines and pro-inflammatory proteins compared to BM-MSCs [54]. In addition, BM-MSCs have been reported to possess better angiogenic bioactivities in vitro than UC-MSCs [28] and may thus be beneficial for the revascularization of islets. Therefore, studies based on a parallel controlled design are needed to compare the integrated effects of the two types of MSCs on T1D.

In our study, subgroup analysis was performed to compare the effects of UC-MSCs and BM-MSCs on the treatment of T1D in both animal models and patients. The two types of MSCs exhibited similar capacities to decrease blood glucose levels and preserve the β-cell function in the NOD mice. The β-cell mass appeared higher in the UC-MSC group than in the BM-MSC group, although the difference was not statistically significant. In the patients with T1D, UC-MSCs and BM-MSCs were also equally efficient in glycaemic control and β-cell preservation. Patients treated with UC-MSCs showed a tendency towards a greater reduction in HbA1c levels and a smaller reduction in FCP levels compared to those treated with BM-MSCs. No difference in the change in the insulin dose or the PCP levels was observed between the two groups. Considering their equivalent efficacy and the advantages of UC-MSCs, it may be more promising to choose UC-MSCs for the application and optimization of MSC therapy for T1D. However, larger randomized trials are needed to confirm the MSC type that is more appropriate for the treatment of T1D.

The mechanism underlying the MSC-mediated remission of T1D is mainly associated with modulating the autoimmune destruction of islets. We further compared the immunomodulatory effects of UC-MSCs and BM-MSCs in the NOD mice. Both UC-MSCs and BM-MSCs significantly attenuated insulitis compared to the control. The mice in the BM-MSC group exhibited relatively lower insulitis scores and fewer islets with severe inflammatory infiltration compared to the UC-MSC group, although the difference was not statistically significant. An imbalance in pathogenic Th17 cells and Tregs is considered to be an important mechanism contributing to the pathogenesis of T1D [55, 56]. We found that both BM-MSCs and UC-MSCs tended to decrease Th17 cells and increase Tregs in the spleen and PLNs of the NOD mice, especially in the mice that received BM-MSCs. However, no significant difference was observed. Consequently, UC-MSCs and BM-MSCs exhibited similar immunomodulatory abilities in the NOD mice, which can partially explain their comparable efficacy.

This study had some limitations. First, the comparative analysis in the patients with T1D was retrospective and observational. Prospective studies with more rigorous research designs are needed to provide more convincing evidence. Second, we only compared the effects of MSCs from the two most widely used sources. In future research, MSCs from other sources should be compared; the potency assays performed in our study can be used as a reference. Moreover, the origin of tissues is only one of the important factors contributing to MSC heterogeneity. Other contributing factors, such as the isolation and production methods, need to be examined to obtain more homogeneous MSC products to improve the therapeutic efficacy.

## Conclusion

This study was the first to address the necessity of and the strategies for defining the optimal source of MSCs for clinical applications in T1D. We demonstrated the equivalent effectiveness of UC-MSCs and BM-MSCs in glycaemic control and β-cell preservation at both the preclinical and clinical levels. However, considering their abundance, non-invasive collection process and higher cell yields, UC-MSCs appear to be more promising than BM-MSCs for future clinical use in the treatment of T1D. Additional studies comparing MSCs from other tissues as well as larger RCTs are still required to determine the optimal source of MSCs for the treatment of T1D.

## Data Availability

The datasets generated and/or analysed during the current study are available from the corresponding author on reasonable request.

## Abbreviations

MSC: Mesenchymal stem cell
UC: Umbilical cord
BM: Bone marrow
AT: Adipose tissue
T1D: Type 1 diabetes
NOD: Non-obese diabetic
STZ: Streptozotocin
PBS: Phosphate-buffered saline
PLN: Pancreatic lymph node
Th1: T helper 1
Th17: T helper 17
Treg: Regulatory T cell
PDL-MSC: MSC derived from periodontal ligament
RCT: Randomized controlled trial
DMEM: Dulbecco’s Modified Eagle Medium
FBS: Foetal bovine serum
mAb: Monoclonal antibody
GADA: Glutamic acid decarboxylase antibody
IA-2A: Protein tyrosine phosphatase antibody
ICA: Islet cell antibody
IAA: Insulin autoantibody
FCP: Fasting C-peptide
PCP: Postprandial C-peptide
SEM: Standard error of the mean
DK: Diabetic ketosis
DKA: Diabetic ketosis acidosis
BMI: Body mass index
HbA1c: Haemoglobin A1c
